# Climatic sensitivity of migraine: a 14-year time series analysis of primary care consultations in Spain

**DOI:** 10.22514/jofph.2026.015

**Published:** 2026-03-12

**Authors:** Juan Nicolás Cuenca-Zaldívar, Carmen Corral del Villar, Silvia García Torres, Rafael Araujo Zamora, Paula Gragera Peña, Nina Cadeau Comte, André Mariz de Almeida, Rob Sillevis, Eleuterio A. Sánchez-Romero, Rosana Cid-Verdejo

**Affiliations:** ^1^Physical Therapy Unit, Primary Health Care Center “El Abajón”, 28231 Las Rozas de Madrid, Spain; ^2^Research Group in Nursing and Health Care, Puerta de Hierro–Segovia de Arana Health Research Institute (IDIPHISA), 28222 Majadahonda, Spain; ^3^Interdisciplinary Research Group on Musculoskeletal Disorders, 28016 Madrid, Spain; ^4^Physical Therapy Unit, Primary Health Care Center “Cerro del Aire”, 28220 Majadahonda, Spain; ^5^Physical Therapy Unit, Primary Health Care Center “San Juan de la Cruz”, 28223 Majadahonda, Spain; ^6^Université Toulouse–Jean Jaurès, 31058 Toulouse, France; ^7^CiiEM—Egas Moniz Center for Interdisciplinary Research, Egas Moniz School of Health & Science, 2829-511 Costa da Caparica, Portugal; ^8^Department of Rehabilitation Sciences, Florida Gulf Coast University, Fort Myers, FL 33965, USA; ^9^Physiotherapy and Orofacial Pain Working Group, Sociedad Española de Disfunción Craneomandibular y Dolor Orofacial (SEDCYDO), 28009 Madrid, Spain; ^10^Faculty of Dentistry, Universidad Complutense de Madrid, 28040 Madrid, Spain; ^11^Department of Clinical Dentistry, Faculty of Biomedical Sciences, Universidad Europea de Madrid, 28005 Madrid, Spain

**Keywords:** Migraine, Climatic factors, Biometeorology, Barometric pressure, Wind direction, Meteorosensitivity, Time-series analysis, Primary care

## Abstract

**Background**: Climatic variability has been proposed as a trigger for migraine; however, evidence from long-term primary care datasets remains scarce. Understanding how atmospheric conditions influence healthcare utilization may improve migraine prediction and management. This study aimed to analyze the association between climatic variables and weekly migraine consultations over a 14-year period in Spanish primary care and to identify the most accurate predictive time-series model. **Methods**: Weekly migraine consultations from 2010 to 2023 were extracted from electronic medical records using the International Classification of Primary Care, Second Edition (ICPC-2) code N89.01. Meteorological variables—temperature, diurnal variability, day-to-day change, wind direction and speed, barometric pressure, and sunshine hours—were obtained from the Spanish State Meteorological Agency (AEMET). Time-series analyses used exponential smoothing state-space models with external regressors (ETSX) and AutoRegressive Integrated Moving Average models with eXogenous regressors (ARIMAX). Model performance was assessed using Root Mean Squared Error (RMSE), Symmetric Mean Absolute Percentage Error (SMAPE), and Mean Absolute Scaled Error (MASE). **Results**: A total of 3176 migraine consultations were identified (mean age 47.6 ± 15.3 years; 81.7% female). The ARIMAX model showed the best predictive performance (RMSE = 3.485, SMAPE = 73.840, MASE = 0.875). Stationarity was confirmed using the Augmented Dickey–Fuller test (*p* = 0.01), and residuals showed no autocorrelation (Ljung–Box test, *p* = 0.833). After multivariable adjustment, female sex was the only variable independently associated with weekly migraine consultations; temperature, barometric pressure, diurnal variability, and wind speed showed no independent effects. Forecasting indicated a stable trend over the subsequent four years. **Conclusions**: This long-term time-series analysis showed that female sex was the only variable independently associated with weekly migraine consultations in primary care. Although most atmospheric indicators did not retain significance, climate-informed ARIMAX modeling improved prediction accuracy and may support personalized, weather-adapted preventive strategies.

## 1. Introduction

Humans interact continuously with their environment, and climatic variability is increasingly recognized as a significant factor influencing human health and disease patterns [1]. Biometeorology, a discipline that studies the interactions between atmospheric conditions and living organisms, provides an essential framework for understanding how climate may modulate physiological responses [2]. Within this field, meteoropathy describes the onset or exacerbation of symptoms triggered by weather changes [3]. It is estimated that approximately 30% of the general population experiences weather-related disorders [4], and up to 75% of patients with chronic pain report symptom fluctuations associated with meteorological factors [5].

Climatic variables, such as barometric pressure, humidity, temperature, and wind fluctuations, can influence several physiological systems involved in migraine susceptibility by modulating neuroendocrine balance, autonomic reactivity, and central sensory processing [3, 6]. These atmospheric transitions may disrupt homeostatic regulation within brainstem circuits—particularly those integrating vestibular and trigeminal inputs—thereby lowering the activation threshold for migraine [7, 8]. Beyond these effects, climatic fluctuations may also modulate additional biological pathways implicated in migraine pathophysiology, including hypothalamic regulation of circadian rhythms, calcitonin gene–related peptide (CGRP)-mediated trigeminovascular activation, and baroreceptor sensitivity to pressure gradients [9, 10]. Together, these mechanisms provide a coherent physiological basis for the precipitation or amplification of migraine susceptibility by external atmospheric stimuli.

Migraine is one of the most prevalent neurological disorders worldwide, affecting an estimated 1.1 billion individuals, approximately 14–15% of the global population, and ranking among the top three causes of disability-adjusted life years (DALYs) in young and middle-aged women [11].

According to the International Classification of Headache Disorders, 3rd edition (ICHD-3), migraine is a primary headache disorder characterized by recurrent attacks of moderate-to-severe pain, often accompanied by photophobia, phonophobia, and nausea [12]. Numerous studies have identified climatic triggers—including sudden drops in barometric pressure, fluctuations in temperature, and changes in humidity or wind speed—as common precipitants of migraine attacks [13, 14, 15]. Epidemiological evidence suggests that variations in atmospheric pressure and temperature can influence both the onset and intensity of migraine episodes [16, 17, 18], whereas seasonal and circadian environmental patterns have been linked to migraine chronobiology [9]. A recent systematic review and meta-analysis confirmed these associations, reporting significant effects of temperature (odds ratio (OR) = 1.15) and ambient pressure (OR = 1.07) on migraine attacks [19]. However, most available data are from short-term or self-reported studies, and few have employed objective long-term primary care datasets to explore these associations systematically [20].

Recent big data and digital health studies have expanded our understanding of the weather–migraine relationship. For example, increased humidity, low barometric pressure, and abrupt wind changes have been correlated with higher rates of migraine attacks in population-based cohorts and smartphone-based tracking systems [21, 22]. A nationwide population study in Asia also demonstrated that temperature variability is significantly associated with migraine onset [23]. These findings highlight the potential impact of meteorological fluctuations on migraine chronobiology and healthcare demand. Nonetheless, inconsistencies persist across studies, suggesting that the direction and magnitude of these associations may vary according to geographical region, population characteristics, and individual meteorosensitivity [18, 20, 24].

Despite growing evidence linking weather to migraine occurrence, no previous study has examined the influence of climatic variables on weekly migraine consultations in primary care over an extended period of time using advanced time-series models with external regressors. Understanding how environmental variability affects migraine-related healthcare utilization may improve patient education, preventive counseling, and resource allocation.

Therefore, the present study aimed to analyze the association between climatic variables, including temperature, precipitation, wind characteristics, sunshine hours, barometric pressure, and the number of weekly primary care consultations for migraine between 2010 and 2023. The secondary objectives were to evaluate the effects of age and sex on these associations, and to describe the temporal trends in migraine consultation rates across the 14-year observation period. Understanding these associations may support preventive strategies and improve health care resource planning.

## 2. Methods

### 2.1 Data source and study population

A retrospective cohort study was conducted using data extracted from the electronic health records of 3176 patients from three primary care centers in Madrid, Spain: “El Abajón” (Las Rozas), “Cerro del Aire” (Majadahonda), and “San Juan de la Cruz” (Pozuelo de Alarcón). All clinical variables were extracted, processed, and analyzed in an anonymized form. Re-identification was not possible at any stage of the study.

The study period extended from 01 January 2010, to 31 December 2023, and included all patients aged 18 years or older who consulted for migraine during this period. Diagnoses were identified using the International Classification of Primary Care, Second Edition (ICPC-2), specifically code N89.01 (Migraine).

Sociodemographic data (age and sex) were retrieved from electronic medical records along with the corresponding consultation date. Meteorological data were obtained from the Spanish State Meteorological Agency (AEMET) station in Pozuelo de Alarcón (ID3194Y; latitude 40°26′54′′N, longitude 3°48′48′′W), located within 15 km of all participating centers. The total catchment area covered approximately 140 km^2^.

This study was approved by the Research Ethics Committee of Puerta de Hierro Majadahonda Hospital (approval code: PI 70/24, Act 06/2024). All the procedures followed the Declaration of Helsinki and ensured complete patient anonymity and confidentiality. This manuscript adheres to the Strengthening the Reporting of Observational Studies in Epidemiology (STROBE) guidelines [25].

### 2.2 Eligibility criteria (inclusion/exclusion)

(a) Population & setting (inclusion): Weekly primary-care consultations between 01 January 2010 and 31 December 2023 from three Madrid centers (“El Abajón”, “Cerro del Aire”, “San Juan de la Cruz”) involving adults ≥18 years with a migraine diagnosis coded in ICPC-2 N89.01. Age and sex were retrieved from electronic medical records.

(b) Exposure (inclusion): Weekly meteorological indicators from the AEMET Pozuelo de Alarcón station for the same period: mean/maximum/minimum temperature, precipitation, wind direction, mean and gust wind speeds, sunshine hours, and maximum/minimum barometric pressure.

(c) Time-series covariates (inclusion): Age, sex, and the aforementioned meteorological variables were considered as external regressors in the ETSX and ARIMAX frameworks. We screened for collinearity and excluded variables with variance inflation factor (VIF) >10. Handling of missingness: weeks with missing series fields (<2% of the total) were handled by Predictive Mean Matching (PMM) prior to modeling, preserving the weekly alignment between clinical and meteorological data.

(d) Exclusion criteria (records): Encounters <18 years; dates outside the study window; encounters without ICPC-2 N89.01; duplicates (same patient, date, and code); and missing data or essential demographics preventing weekly aggregation.

(e) Handling of missingness: Weeks with missing series fields (≤2% of the total) were imputed using Predictive Mean Matching (PMM) to preserve alignment between clinical and meteorological series.

(f) Outcome definition: The primary outcome was the number of weekly migraine consultations (ICPC-2 N89.01). Descriptive characteristics are reported in **Supplementary Table 1**, the final model summary in Table 1, and model selection in **Supplementary Table 2**.

**Table 1.** Migraine final time-series model summary.

	Coefficient (SE)	95% CI	^a^*p* value
Age	−0.021 (SE = 0.016)	−0.052, 0.01	*Z* = −1.34, *p* = 0.18
Gender (Female)	0.204 (SE = 0.093)	0.022, 0.386	*Z* = 2.20, ***p* = 0.028**
Gender (Male)	−0.163 (SE = 0.179)	−0.514, 0.188	*Z* = −0.911, *p* = 0.362
Average temperature (degrees Celsius)	−0.001 (SE = 0.024)	−0.049, 0.046	*Z* = −0.057, *p* = 0.955
Diurnal temperature range (degrees Celsius)	−0.063 (SE = 0.06)	−0.18, 0.054	*Z* = −1.059, *p* = 0.289
Day-to-day temperature change (degrees Celsius)	−0.085 (SE = 0.17)	−0.418, 0.248	*Z* = −0.501, *p* = 0.616
Average rainfall (L/m^2^)	−0.018 (SE = 0.051)	−0.118, 0.082	*Z* = −0.349, *p* = 0.727
Average wind speed (m/s)	−0.357 (SE = 0.223)	−0.794, 0.079	*Z* = −1.604, *p* = 0.109
Wind gusts (m/s)	0.106 (SE = 0.11)	−0.11, 0.322	*Z* = 0.963, *p* = 0.336
Sunshine hours	0.071 (SE = 0.087)	−0.099, 0.242	*Z* = 0.822, *p* = 0.411
Average barometric pressure (hPa)	0.135 (SE = 0.104)	−0.068, 0.339	*Z* = 1.303, *p* = 0.193
Day-to-day barometric pressure change (hPa)	0.199 (SE = 0.212)	−0.216, 0.613	*Z* = 0.939, *p* = 0.348
Wind direction (cosine)	6.404 (SE = 1.772)	2.929, 9.878	*Z* = 3.613, ***p* < 0.001**
Wind direction (sine)	3.056 (SE = 1.776)	−0.425, 6.538	*Z* = 1.721, *p* = 0.085
Age (Male)	0.006 (SE = 0.004)	−0.001, 0.014	*Z* = 1.675, *p* = 0.094
Age (Female)	0.002 (SE = 0.002)	−0.002, 0.006	*Z* = 1.088, *p* = 0.276

*SE: Standard Error; 95% CI: 95% confidence interval. ^a^significant at p < 0.05 (shown in bold). The gender (female) and gender (male) coefficients correspond to deviation coding (sum-to-zero contrasts), which does not introduce collinearity with the intercept. Females correspond to +0.5, and males to −0.5 deviation weights. Therefore, both coefficients appear, but the reference is the grand mean rather than a single sex category.*

### 2.3 Climatic variables

Meteorological data corresponding to the same geographical region and timeframe were obtained from the Spanish State Meteorological Agency (AEMET). The variables collected included the following: mean temperature (°C), diurnal temperature range (°C), day-to-day temperature change (°C), wind direction (°), mean and gust speed (m/s), mean barometric pressure and day-to-day change (hPa), and sunshine hours (h/day). In the case of wind direction, because it is a circular variable where North is both at 0° and 365°, it was decomposed into cosine and sine components to account for circularity. Both components were included in the model; however, only the cosine term remained significant in the final ARIMAX model.

Daily data were aggregated into weekly averages to match the temporal resolution of medical consultations, which is necessary to obtain a complete time series without missing data, and is a standard approach in time-series analysis [26]. These indicators were selected based on prior evidence suggesting that variations in temperature, barometric pressure, and wind patterns can influence migraine onset and severity through neurovascular and autonomic mechanisms [10, 16, 27].

### 2.4 Follow-up process for consultations

All primary care migraine consultations recorded between 2010 and 2023 were monitored to construct a weekly time-series of migraine cases. This longitudinal dataset enabled the analysis of temporal fluctuations, stationarity, and recurrence patterns in healthcare utilization.

Linking demographic and meteorological variables allowed for the evaluation of environmental influences on migraine incidence in real-world primary care settings.

### 2.5 Sample size

According to the criteria of Burmeister *et al*. [28], a minimum of 240 observations is required for adequate statistical power in a multiple regression model with 11 continuous predictors and one dichotomous variable.

The present study far exceeded this threshold, comprising 3176 migraine consultations over 14 years. Weekly data aggregation yielded approximately 728 time-series observations, providing robust statistical power for both the exponential smoothing state-space models with external regressors (ETSX) and AutoRegressive Integrated Moving Average models with eXogenous regressors (ARIMAX) modeling.

### 2.6 Study procedures

Patient data, including age, sex, and ICPC-2 diagnostic codes, were anonymized and extracted from the electronic medical records. These clinical variables were systematically linked to the corresponding meteorological parameters obtained from the Spanish State Meteorological Agency (AEMET) during the same observation period. The climatic dataset included mean temperature (°C), diurnal temperature range (°C), day-to-day temperature change (°C), wind direction (°) transformed into cosine and sine of wind direction, mean and gust speed (m/s), mean barometric pressure and its day-to-day change (hPa), and sunshine hours (h/day) [26].

Each variable was aggregated on a weekly basis to align with the frequency of medical consultations and ensure consistency between environmental exposure and health care activities. The integrated dataset captured the joint variability of clinical, demographic, and climatic factors relevant to migraine occurrence.

### 2.7 Outcome measures

The primary outcome was the weekly number of primary care consultations for migraine, as identified by ICPC-2 codes.

Secondary analyses explored the effects of sex, age, and climatic variables on temporal consultation trends, and evaluated potential seasonal or environmental triggers influencing migraine frequency. Diagnostic criteria were consistently applied across the 14-year period, and meteorological variables were defined according to the AEMET operational standards to ensure homogeneity and reproducibility across all data sources.

### 2.8 Statistical analysis

Statistical analyses were performed using R version 4.1.3 (R Foundation for Statistical Computing, Vienna, Austria). The significance level was set at *p * < 0.05. Quantitative variables are presented as mean ± standard deviation and qualitative variables as absolute and relative frequencies (%).

A weekly time series of migraine cases from 01 January 2010, to 31 December 2023, was analyzed, incorporating external regressors: age, sex, mean temperature, diurnal temperature range, day-to-day temperature change, wind direction, mean speed, and gust speed, as well as mean barometric pressure and its day-to-day change, eliminating variables with a variance inflation factor (VIF) greater than 10. In the case of wind direction, because it is a circular variable where North is both at 0° and 365°, it was segmented into the cosine and sine of the wind direction, being considered significant only if both had a significant effect simultaneously [29, 30]. In addition, we explored the interaction between age and gender, adding the interaction terms age:gender (male) and age:gender (female) to the model.

Model selection between the exponential smoothing state-space model with external regressors (ETSX) and AutoRegressive Integrated Moving Average models with eXogenous regressors (ARIMAX) was based on predictive accuracy, which was evaluated using a training set (75%) and a test set (25%). The models were compared using the Root Mean Squared Error (RMSE), Symmetric Mean Absolute Percentage Error (SMAPE), and Mean Absolute Scaled Error (MASE), with lower values indicating a better fit [31, 32].

The stationarity of the series was assessed using the augmented Dickey (ADF) test. Residual autocorrelation was evaluated using the Ljung-Box test, and residual normality was tested using the Kolmogorov-Smirnov test with Lilliefors correction.

For 14 weeks (1.92% of the series) with missing data on age, sex, and migraine, imputation was performed using the predictive mean matching (PMM) method implemented in the mice package in R [33].

All statistical procedures were double-checked for reproducibility and internal consistency. This methodology ensures robust modeling of migraine case time series, appropriately accounting for external factors, handling missing data, and validating model assumptions.

## 3. Results

A total of 3176 migraine cases were included in the analysis, with a mean age of 47.59 ± 15.29 years and a predominance of female patients (81.71%) (**Supplementary Table 1**).

A generally stable temporal trend was observed in the number of migraine consultations throughout the 14-year study period, with a slight decrease in the number of consultations in 2015 (Fig. 1).

**Fig. 1.** Weekly number of migraine consultations from January 2010 to December 2023 in the three participating primary care centers. The series displays both short-term fluctuations and stable long-term patterns over the 14-year period.

All 16 explanatory variables were retained after verifying the absence of multicollinearity (variance inflation factor of <10). Model comparison identified the ARIMAX model (AR0, I0, MA2, SAR0, SI0, SMA0, LAG1, and m52) as the best-fitting structure for migraine prediction (**Supplementary Table 2**).

The Augmented Dickey-Fuller test confirmed stationarity in the series (*p* = 0.01). Residual diagnostics revealed no autocorrelation (Ljung-Box test, *p* = 0.833), but a non-normal distribution (Kolmogorov-Smirnov test, *p* = 0.009).

Within the migraine process, only female sex was positively associated (*p* = 0.028) (Table 1).

Forecasting for the subsequent four-year horizon indicated a stable trend in the expected number of migraine consultations, without substantial seasonal peaks (Fig. 2).

**Fig. 2.** Four-year ARIMAX forecast of weekly migraine consultations using the best-fitting model (AR0, I0, MA2, LAG1, seasonal period m = 52). The solid line represents projected values and the shaded band represents 95% confidence intervals.

## 4. Discussion

### 4.1 Principal findings

This 14-year time-series analysis demonstrated that female sex was the only variable significantly associated with weekly migraine consultations in Spanish primary care. Contrary to earlier hypotheses and some prior studies, temperature, barometric pressure, diurnal variability, wind speed, and pressure-change indicators did not remain significant predictors after multivariable ARIMAX modeling.

The ARIMAX model showed superior predictive performance compared with ETSX, confirming the value of incorporating external regressors even when only a limited number exhibited independent effects. The overall temporal pattern of migraine consultations was stable across the study period, with no long-term upward or downward trend.

Importantly, components of wind direction showed partial directional asymmetry, suggesting that specific airflow patterns or transitions between air masses may modulate the weekly migraine burden. Female sex also showed a positive association with consultation frequency, consistent with the recognized higher vulnerability of women to migraine, but observed here specifically in healthcare utilization, rather than as a pure prevalence signal.

### 4.2 Comparison with previous studies

Our findings partially align with previous epidemiological and biometeorological research showing that climatic dynamics can influence migraine occurrence [9, 13, 14, 15, 16, 17, 18, 20, 21, 22, 23]. Numerous studies have described associations between migraine and temperature, barometric pressure, or humidity, although these associations are inconsistent across populations and methodologies [13, 14, 15, 16, 17, 18, 21, 22, 34, 35].

In contrast to many earlier reports, temperature and barometric pressure were not independently associated with migraine consultations in our adjusted models. Similar null findings have been documented in large administrative datasets, including the recent Japanese cohort by Tatsumoto *et al*. [18], who found no direct link between atmospheric pressure variability and migraine incidence despite strong seasonal atmospheric changes.

However, our study confirms that wind-related variables remain relevant, in line with smartphone-based analyses and artificial intelligence (AI)-driven approaches showing that wind speed, direction, and frontal transitions influence migraine patterns [20, 21]. The inverse association found here suggests that certain predominant wind patterns, possibly associated with dry air, descending air masses, or pressure-front stabilization of atmospheric fronts, could modulate the trigeminovascular activation thresholds.

Regarding sex differences, our findings reinforce established knowledge that women consistently show higher sensitivity to environmental fluctuations, likely mediated by hormonal, circadian, and trigeminovascular interactions [13, 23]. Female sex was the only demographic predictor retained in the final model that aligns with epidemiological prevalence patterns and corrects the earlier interpretation prior to refined modeling.

### 4.3 Mechanistic considerations

The biological plausibility of wind-related effects on migraines aligns with several mechanistic pathways. Airflow and directional changes often coincide with shifts in atmospheric stability, humidity, ion concentration, and barometric gradients. Such transitions may influence baroreceptor sensitivity, vascular tone and cerebral perfusion, trigeminal nociceptive signaling, and autonomic-endocrine regulation [10, 27]. Animal research provides further support: exposure to falling barometric pressure activates the vestibular and trigeminal nuclei, lowering the threshold for migraine onset [7]. Moreover, wind-driven changes can modulate mucosal irritation, central sensory processing, and premonitory symptom expression—elements consistent with established migraine neurobiology [10, 13]. The absence of independent effects for temperature, pressure, and humidity after multivariable adjustment suggests that wind direction may act as a proxy marker for broader meteorological transitions rather than as a direct isolated trigger.

### 4.4 Clinical and public health implications

From a clinical standpoint, these findings highlight the relevance of integrating biometeorological information—particularly wind-related indicators—into migraine management. Although most climatic variables were not independent predictors in the final ARIMAX model, the partial directional asymmetry observed in wind components suggests that climate-sensitive predictive tools could still assist clinicians in anticipating weeks of increased migraine consultations. This may support timely adjustment of preventive or symptomatic treatments and facilitate personalized recommendations, such as optimizing hydration, limiting outdoor exposure during abrupt meteorological transitions, or adapting work routines for patients with documented meteorosensitivity [34].

On a broader scale, the integration of meteorological forecasting into primary care surveillance systems could serve as an early warning mechanism, similar to models already used in respiratory and cardiovascular medicine [27]. Considering that migraine is one of the leading causes of neurological disability and primary care visits worldwide [11], even minor reductions in weather-related exacerbations could translate into substantial healthcare savings and improved quality of life [35].

### 4.5 Limitations and future directions

This study had several limitations. Using a single meteorological station restricts spatial resolution and may not capture microclimatic variability [35]. Although sex was included as a covariate in the ARIMAX model, the structure of the electronic health record dataset did not allow sex-stratified analyses. This limitation restricts the ability to determine whether climatic effects differ between men and women and should be considered when interpreting the generalizability of the findings. Other environmental cofactors, including air pollution, aeroallergens, and noise, were not analyzed, although evidence suggests that they may interact with meteorological dynamics to influence migraine risk [21, 34, 36]. Information theory-based analyses show that migraine triggers frequently involve overlapping and non-linear interactions rather than isolated exposures [14], reinforcing the need for more sophisticated modelling of environmental determinants. Previous studies combining meteorological and pollution data have shown synergistic effects on headache burden [36], underscoring the importance of incorporating these exposures into future biometeorological models.

Moreover, the electronic health records did not capture key clinical migraine variables, such as attack frequency, pain intensity, presence of aura, or acute and preventive medication use, which restricts interpretations to consultation-based outcomes rather than symptom-level dynamics. This lack of clinical granularity also prevents the assessment of individual meteorosensitivity profiles or medication-related modifiers of climatic effects.

Future research should integrate multi-station meteorological datasets, wearable technology, ecological momentary assessment, machine learning approaches for non-linear exposure–response patterns, and circadian, hormonal, and psychosocial moderators. Prospective biometeorology–neurology designs are essential to identify which patients are truly meteorosensitive and through which mechanisms.

### 4.6 Ethical considerations in the context of clinical care

The findings of this study illuminate an often-overlooked ethical dimension rooted in patients’ embodied encounters with their environment. The recurrent associations between climatic variables and migraine consultations underscore how patients develop a nuanced and situated awareness of their personal meteorosensitivity. This lived experience fosters a form of empowerment that transcends clinical prescriptions: patients actively interpret and respond to weather-related cues, effectively territorializing their own care within a geographic and temporal landscape shaped by atmospheric dynamics.

Such territorialization implies profound ethical engagement with migraine management, positioning the patient as a co-creator of their therapeutic context. Rather than being mere subjects of clinical observation, patients become attuned agents whose weather-informed self-monitoring complements and enriches biomedical frameworks. This dynamic challenges traditional hierarchies in clinical care by valuing experiential knowledge along with the statistical and model-based predictions presented in this research.

Moreover, this interplay between lived experience and scientific insight invites a reconfiguration of ethical care practices that respect patient autonomy while recognizing the interdependencies between the body, environment, and healthcare systems. Clinicians are, thereby, called to honor this situated expertise, facilitating collaborative dialogue that bridges meteorological data with the patient’s environmental and sensory realities. Ultimately, this ethical stance promotes more responsive, personalized migraine care that embraces complexity and fosters resilience through patient empowerment within their lived climatic territory.

## 5. Conclusions

This 14-year time-series analysis of primary care data showed that only female sex was independently associated with weekly migraine consultations, whereas temperature, barometric pressure, humidity, diurnal variability, wind speed, and pressure change indicators did not retain significance after multivariable ARIMAX adjustment.

Components of wind direction showed partial directional asymmetry, with the cosine term reaching statistical significance; however, the absence of a fully significant circular effect precludes concluding an independent association of wind direction as a whole. These findings refine previous assumptions about weather-related migraine triggers and suggest that airflow patterns and transitions between air masses, rather than absolute climatic values, may play a more relevant role in modulating short-term migraine burden.

The ARIMAX model provided the most accurate predictive performance, confirming the value of incorporating external regressors, even when only a limited subset exerts an independent effect. Forecasting revealed a stable pattern of migraine consultations over the next four years, without marked seasonal peaks.

Overall, these results support the concept of migraine meteorosensitivity, while emphasizing that its expression may be more subtle and variable than traditionally assumed. These findings must be interpreted with caution given the absence of clinical details (*e.g.*, attack severity, aura, and medication use) in primary care records, which limits analyses to consultation frequency rather than symptom burden. Integrating wind-related indicators into clinical counseling and predictive tools could improve the anticipation of high-risk weeks and support personalized, climate-informed preventive strategies, potentially enhancing patient self-management and primary care resource planning.

These conclusions should also be interpreted considering the study’s broader limitations, particularly the absence of important environmental and behavioral confounders (such as air pollution, stress, and seasonal infections), which could not be incorporated owing to the constraints of the electronic health record system. Although sex was included as a covariate, the dataset structure did not allow for sex-stratified analyses, which limited the exploration of potential sex-specific climatic effects. Future studies integrating richer clinical data, multi-station meteorological sources, and advanced analytical approaches may help to characterize individual meteorosensitivity more precisely and strengthen the clinical applicability of climate-informed predictive models.

## Supplementary Material

Supplementary material associated with this article can be found, in the online version, at https://files.jofph.com/ files/article/2014258846246027264/attachment/ Supplementary%20material.docx.


