# Supplementary material for: Climatic sensitivity of migraine: a 14-year time series analysis of primary care consultations in Spain
Source: J Oral Facial Pain Headache. 2026 Mar 12;40(2):22–30. doi: 10.22514/jofph.2026.015 (PMC13036618; doi:10.22514/jofph.2026.015)
Supplement: Supplementary file 1 [file Supplementary-material.docx]

Supplementary material

Supplementary Table 1. Sample characteristics and atmospheric conditions.

|  | | Overall | 2010 | 2011 | 2012 | 2013 | 2014 | 2015 | 2016 | 2017 | 2018 | 2019 | 2020 | 2021 | 2022 | 2023 |
| --- | --- | --- | --- | --- | --- | --- | --- | --- | --- | --- | --- | --- | --- | --- | --- | --- |
| n | | 3176 | 239 | 288 | 252 | 225 | 200 | 172 | 186 | 240 | 234 | 248 | 214 | 208 | 210 | 260 |
| Socio-demographic characteristics | | | | | | | | | | | | | | | | |
|  | Age | 47.59 ± 15.29 | 52.62 ± 16.26 | 52.01 ± 13.44 | 51.89 ± 15.43 | 51.03 ± 14.32 | 49.56 ± 16.20 | 50.35 ± 14.83 | 47.16 ± 14.97 | 47.86 ± 12.70 | 48.52 ± 13.30 | 42.35 ± 16.70 | 44.50 ± 16.00 | 42.22 ± 12.68 | 43.59 ± 16.22 | 41.88 ± 13.97 |
|  | Gender (Female), n (%) | 2595 (81.71) | 195 (81.59) | 244 (84.72) | 200 (79.37) | 186 (82.67) | 158 (79) | 144 (83.72) | 160 (86.02) | 188 (78.33) | 190 (81.2) | 218 (87.9) | 174 (81.31) | 174 (83.65) | 160 (76.19) | 204 (78.46) |
|  | Gender (Male), n (%) | 581 (18.29) | 44 (18.41) | 44 (15.28) | 52 (20.63) | 39 (17.33) | 42 (21) | 28 (16.28) | 26 (13.98) | 52 (21.67) | 44 (18.8) | 30 (12.1) | 40 (18.69) | 34 (16.35) | 50 (23.81) | 56 (21.54) |
| Atmospheric conditions | | | | | | | | | | | | | | | | |
|  | Average temperature (degrees Celsius) | 14.99 ± 7.66 | 14.74 ± 8.41 | 15.53 ± 7.36 | 14.13 ± 7.91 | 13.77 ± 7.73 | 14.91 ± 6.74 | 15.28 ± 7.74 | 14.73 ± 7.61 | 15.28 ± 7.87 | 14.57 ± 7.80 | 15.12 ± 7.38 | 15.07 ± 7.37 | 14.95 ± 7.29 | 16.14 ± 7.93 | 15.69 ± 7.77 |
|  | Average rainfall (L/m²) | 1.43 ± 4.84 | 2.02 ± 5.44 | 1.26 ± 3.66 | 1.09 ± 4.40 | 1.41 ± 4.48 | 1.48 ± 4.46 | 0.92 ± 3.21 | 1.84 ± 4.97 | 0.85 ± 3.23 | 1.94 ± 5.46 | 1.26 ± 4.91 | 1.40 ± 4.39 | 1.28 ± 4.74 | 1.48 ± 4.22 | 1.79 ± 8.18 |
|  | Average wind speed (m/s) | 2.87 ± 1.77 | 2.71 ± 1.77 | 2.54 ± 1.61 | 2.91 ± 1.84 | 2.90 ± 1.90 | 3.09 ± 1.88 | 2.68 ± 1.72 | 2.73 ± 1.92 | 2.55 ± 1.75 | 3.01 ± 1.71 | 3.29 ± 1.91 | 2.96 ± 1.75 | 2.94 ± 1.60 | 2.83 ± 1.54 | 3.01 ± 1.73 |
|  | Wind gusts (m/s) | 10.03 ± 3.64 | 10.09 ± 3.55 | 9.67 ± 3.32 | 10.26 ± 3.60 | 10.52 ± 3.57 | 10.60 ± 3.59 | 9.72 ± 3.74 | 9.89 ± 3.67 | 9.94 ± 3.67 | 10.10 ± 3.49 | 10.58 ± 4.07 | 9.75 ± 3.79 | 9.90 ± 3.47 | 9.92 ± 3.46 | 9.56 ± 3.82 |
|  | Sunshine hours | 8.20 ± 3.98 | 7.79 ± 4.26 | 8.30 ± 4.05 | 8.47 ± 3.92 | 8.16 ± 4.02 | 8.10 ± 3.97 | 8.31 ± 3.77 | 7.97 ± 4.17 | 8.75 ± 3.59 | 8.05 ± 3.95 | 8.82 ± 3.78 | 7.89 ± 4.20 | 7.86 ± 3.93 | 7.93 ± 4.26 | 8.46 ± 3.57 |
|  | Diurnal temperature range (degrees Celsius) | 13.74 ± 4.93 | 10.94 ± 3.82 | 12.90 ± 4.51 | 14.62 ± 5.10 | 13.68 ± 4.85 | 13.28 ± 4.80 | 13.99 ± 4.79 | 13.79 ± 5.25 | 15.47 ± 4.60 | 13.29 ± 4.73 | 14.47 ± 5.15 | 13.38 ± 5.08 | 13.95 ± 4.92 | 13.90 ± 4.93 | 14.64 ± 4.97 |
|  | Day-to-day temperature change (degrees Celsius) | 0.00 ± 1.97 | 0.01 ± 2.05 | −0.01 ± 1.77 | −0.01 ± 2.03 | 0.01 ± 2.04 | 0.01 ± 1.81 | 0.00 ± 2.08 | −0.02 ± 1.85 | 0.02 ± 2.15 | 0.00 ± 1.98 | 0.00 ± 2.05 | -0.01 ± 2.01 | 0.02 ± 1.99 | 0.00 ± 1.91 | −0.01 ± 1.87 |
|  | Average barometric pressure (hPa) | 4.38 ± 2.30 | 4.95 ± 2.85 | 4.11 ± 1.91 | 4.21 ± 1.81 | 4.60 ± 2.65 | 4.43 ± 2.76 | 4.36 ± 2.22 | 4.52 ± 2.29 | 4.44 ± 2.46 | 4.56 ± 2.48 | 4.57 ± 2.45 | 4.27 ± 2.11 | 4.11 ± 1.98 | 4.15 ± 1.72 | 4.08 ± 2.04 |
|  | Day-to-day barometric pressure change (hPa) | 0.00 ± 2.71 | −0.01 ± 3.34 | 0.01 ± 2.39 | 0.00 ± 2.24 | 0.01 ± 3.03 | 0.00 ± 2.95 | 0.01 ± 2.67 | −0.01 ± 2.81 | 0.02 ± 2.79 | −0.02 ± 2.91 | 0.00 ± 2.89 | 0.00 ± 2.62 | 0.00 ± 2.36 | 0.01 ± 2.26 | 0.00 ± 2.46 |
|  | Wind direction (cosine transformation) | 0.89 ± 0.22 | 0.93 ± 0.05 | 0.94 ± 0.05 | 0.94 ± 0.05 | 0.94 ± 0.05 | 0.94 ± 0.04 | 0.94 ± 0.05 | 0.94 ± 0.05 | 0.92 ± 0.14 | 0.89 ± 0.23 | 0.86 ± 0.27 | 0.83 ± 0.33 | 0.78 ± 0.38 | 0.82 ± 0.35 | 0.81 ± 0.33 |
|  | Wind direction (sine transformation) | 0.34 ± 0.20 | 0.32 ± 0.15 | 0.32 ± 0.14 | 0.32 ± 0.15 | 0.31 ± 0.16 | 0.32 ± 0.14 | 0.31 ± 0.15 | 0.31 ± 0.15 | 0.31 ± 0.17 | 0.35 ± 0.20 | 0.37 ± 0.21 | 0.37 ± 0.25 | 0.41 ± 0.27 | 0.38 ± 0.26 | 0.42 ± 0.24 |

Data expressed with mean ± standard deviation or with absolute and relative values (%). Wind direction was decomposed into sine and cosine transformations to properly account for its circular nature.

Supplementary Table 2. Migraine time-series model selection.

|  | Model parameters | RMSE | SMAPE | MASE |
| --- | --- | --- | --- | --- |
| ETSX model | M, N, N | 15.959 | 83.201 | 1.913 |
| ARIMAX model | AR0, I0, MA2, SAR0, SI0, SMA0, LAG1, m52 | 3.485 | 73.840 | 0.875 |

Models notation: ETSX (error, trend, seasonality), letters M: Multiplicative and N: Non-present. ARIMAX: AutoRegressive Integrated Moving Average models with external regressors; AM: AutoRegressive order; I: Integrated order; MA: Moving Average order; S: Seasonal order component; m: Seasonal period; LAG order; RMSE: Root mean squared error; SMAPE: Symmetric Mean absolute percentage error; MASE: Mean absolute scaled error.
